# Clinical Report on Dysentery

**Published:** 1853-08

**Authors:** Austin Flint


ART. II.— *Clinical Report on Dysentery. Based on an analysis of forty-nine recorded cases.* By AUSTIN FLINT, M. D.

*" C'est en interrogeant fréquemment la nature que nous lui arrachons ses secrets."*

(Continued from page 115.)

SECTION SECOND.

*Circumstances attending the access of the disease.*

By the circumstances attending the access, it is intended to refer to symptoms occurring before the nature of the disease was determinable. The characteristic evacuations, *i. e.* blood and mucus, generally, or the latter alone, constitute the evidence of the existence of dysentery. Until these diagnostic criteria are observable, the affection has not become fully developed. This statement may not be correct in a strict pathological sense; but whether it be or be not true is of no consequence so far as concerns present inquiries. It is plain that, practically, no other course can be pursued. The precedence of ordinary diarrhœa is almost the only point connected with the access on which the histories contain any information. The facts with respect to this point in thirty-three cases were noted. Of these thirty-three cases, diarrhœa preceded the occurrence of dysenteric discharges, for a greater or less period, in thirty cases, and the characteristics of dysentery were stated to have been manifested at the commencement of illness in only three cases.

The duration of the antecedent diarrhœa was ascertained in twenty-seven cases. In four cases the dysenteric discharges appeared within twenty-four hours from the time that the patients were attacked with diarrhœa. They made their appearance on the second day in eleven cases; on the third day in five cases; on the fourth day in three cases; on the fifth day in one case; on the seventh day in one case, and in two cases it is recorded that several days intervened.

In several instances the preliminary diarrhœa occurred at night. The dysenteric characters, also, in several cases first occurred during the night.

Of the fatal cases, the facts with respect to antecedent diarrhœa are noted in six only. In each of these cases diarrhœa preceded; in one case for twelve hours, in one case for one day, in two cases for two days, in one case for three days, and in one case the duration was not ascertained.

Diarrhœa is the only event of importance noted in most of the histories to have attended the development of the disease.

### SECTION THIRD.

### *Symptoms referable to the digestive system.*

*Alvine dejections.* The discharges from the bowels, of course, in all the cases, were abnormally frequent, and presented, during the progress of the disease, blood and mucus, more or less in quantity. The appearances, however, were not uniform. Considerable differences existed in the different cases, and often in the same case at different times. The more important of the varieties, so far as concerns the characters peculiar to dysentery, may be distributed in the following classes:

1. Mucus, usually more or less commingled with blood, the quantity expelled, at a time, generally small, but occasionally more abundant, forming a jelly-like mass.

2. Fibrinous laminæ, or small flakes distinguished by their firmness and opacity; often more or less tinged with blood, occasionally having a greenish tint; varying in quantity, but generally more copious than the former.

3. Puruloid matter. This was observed in a very few instances only.

4. Sero-sanguinolent fluid; oftener copious, than small in quantity.

The foregoing forms of dysenteric discharges were frequently more or less combined, or appeared in succession, and sometimes in alternation in the same case. Evacuations often consisted wholly of these morbid products. Sometimes they were mixed with fecal matter, the latter presenting various appearances, and in several instances, during the progress of the disease, dejections entirely fecal, and more or less abundant, took place, and were followed by discharges with the dysenteric characters not less than before. Feces in small solid lumps, (scybala,) were noticed but in a few instances. The feculent discharges varied in consistence and color, being sometimes green, in other instances yellow, and occasionally brown. Several times consistent moulded feces were evacuated, preceded and followed by dysenteric discharges. In the proportionate quantity of fecal matter, as well as in the infrequency of its appearance in the discharges, different cases differed greatly.

Occasionally small dejections entirely hemorrhagic occurred, taking place immediately, or shortly after discharges of a different character, the blood evidently escaping from the vessels of the rectum near the anus.

This is a brief summary of the diseased appearances which the alvine dejections presented. To determine the numerical ratio which these appearances severally bore to each other, would not subserve any useful end, and, moreover, it would be impossible from the data contained in the histories, inasmuch as the precise number of evacuations, with their characters individually were in no instance fully recorded.

The chief point of interest pertaining to the variations in the evacuations from the bowels, aside from the importance which belongs to these symptoms as elements of the natural history of the disease, is this:—what pathological significance is to be attached to them respectively? With respect to this inquiry, an examination of the facts relating to the subject points to certain conclusions.

1. The frequency of the dejections is no criterion of the gravity and danger of the disease. In this particular the cases differed greatly, evacuations occurring in some instances hourly, or even half-hourly, and in other instances several hours intervening between them. Cases also differed in this respect at different times in the progress of the disease. In the case in which the largest number, (sixty,) of dejections were noted to have occurred in the course of twenty-four hours, recovery took place; and, as it happens, also, in a case in which there were as small a number throughout the disease as in any other, the issue was fatal. The average number of dejections in the fatal cases would fall short of the average number in the cases which ended in recovery.

2. Fibrinous *laminæ*, or flakes, occurred generally in the fatal cases, and this character was oftener present in severe, than in mild cases.

3. Other things being equal, cases appeared to be grave in proportion to the copiousness of the discharges having the character just referred to, and with a proportionably small quantity of fecal matter.

4. The sero-sanguinolent discharge indicates, more than any other kind of evacuation, gravity and danger, and its significance is proportionate to the quantity expelled. This character of dejection occurred in nearly all the fatal cases, and in but a small proportion of those in which recovery took place. To enumerate with respect to this fact, eight of the eleven fatal cases were characterized by the evacuation of a sero-sanguinolent liquid in greater or less abundance, while in only four of the remaining thirty-eight cases is this kind of dejection noted, and in these four cases the quantity was not large. By the sero-sanguinolent discharge I mean a thin, bloody fluid, compared by writers to the appearance of water in which meat has been washed, (lotura carnium.) The effect of a copious sero-sanguinolent evacuation as indicated by the pulse was strikingly illustrated in one of the cases. At 9½, P. M., in this case, the pulse numbered 120; at 11½, P. M., a copious sero-sanguinolent dejection having in the mean time taken place, the pulse had risen to 135.

5. The morbid appearances pertaining to the alvine dejections, may be slight in cases in which the general symptoms denote great gravity, and which end fatally. This fact was strikingly exemplified in a case in which the dejections at no time contained more than a small amount of blood and mucus, and were never frequent, averaging only four or five in the twenty-four hours. In this case, the dejections for several days prior to the day before death, were entirely free from any of the dysenteric characters. This was one of the cases occurring in 1849, when epidemic cholera prevailed to a considerable extent. The discharges, however, did not present the choleraic characters. No case of this description was observed when cholera did not prevail, and in farther support of the supposition that the disease in these cases derives its severity and fatality from the combination of the unknown internal pathological condition which obtains in cholera, with the dysenteric affection, it may be mentioned that during the prevalence of the former, cases occasionally occur in which the state of collapse and death by asthenia take place when the dejections are free both from the choleraic and dysenteric sensible characters. A few such cases have fallen under my observation, during the prevalence of cholera, and at no other time.

The occurrence of sero-sanguinolent dejections, also, it may be suspected, had some connection with the prevalence of cholera. In not one of the twelve cases collected prior to 1849, was this symptom noted, but it occurred in some of the cases in 1850 and 1851, when cholera prevailed only to a small extent. It is probable that this feature of dysentery is oftener present when the disease prevails as an epidemic than in sporadic cases. With reference to these points a larger number of observations is desirable.

The examination of the alvine dejections in all the cases recorded, was limited to the gross appearances. A series of observations with the aid of chemical analysis and the microscope, would doubtless be interesting, and might possibly lead to important pathological deductions.

*Tormina and tenesmus.* These symptoms were generally present, but in the degree of suffering therefrom, the cases differed considerably. Their prominence was not in proportion to the gravity of the disease. On the contrary, they were most marked in cases which, so far as regards the indications of danger were mild.* In the majority of the fatal cases they had little prominence, and, indeed, in some cases among those characterized by sero-sanguinolent discharges, they were scarcely present. This is the only point of interest developed by an examination of the facts pertaining to these symptoms which the histories contain.

*Vomiting.* This symptom is noted in the histories of several cases, and in the remainder nothing is stated respecting its presence or absence. It may have occurred in more or less of these cases, but could not have been prominent. The number of cases in which it is noted to have occurred, is twelve. In most of these cases it was not very prominent, that is to say it was not urgent and persisting. So far as we may judge from this collection of cases, vomiting does not occur in a notable degree in the majority of cases of dysentery. It occurs oftener, and is more marked, in cases distinguished for their gravity. Thus of the twelve cases in which it is noted to have occurred, six were fatal cases. This is relatively a large proportion comparing the number of fatal cases, (eleven,) with the number of those ending in recovery, (thirty eight.) Making due allowance for incompleteness of the

---

* Thisfact is directly opposed to the statement by Valleix, viz., that the tenesmus proportion to the gravity of the disease.

histories as respects this symptom, the statement just made seems admissible. The presence of this symptom, therefore, should affect unfavorably, to a certain extent, the prognosis.

*Abdominal tenderness.* The histories contain information as to abdominal tenderness in thirty-three cases. Of these cases this symptom was altogether absent, or scarcely appreciable in fourteen, and present, in a greater or less degree, in nineteen.

When present, it was not a prominent symptom except in a few instances. It was slight, or moderate in degree, in ten, and considerable in five cases. In a single case only was the tenderness great.

In nine cases the situation of the tenderness is mentioned. It existed over the entire abdomen in two cases; over the tract of the transverse colon in one case; over the transverse and descending colon in one case; in the left iliac region in two cases; in the right iliac region in one case, and over the tract of the colon in one case, and over the lower part of abdomen in one case.

Of the fatal cases, the presence or absence of tenderness was noted in six; and of these six cases it was present in two, and absent in four. This last result is interesting, tending to show that abdominal tenderness is oftener present in cases which recover, than in those ending fatally, a conclusion which probably would not have been anticipated.

These enumerations suffice to prove that abdominal tenderness is not a constant, but only a tolerably frequent event in the natural history of dysentery, being almost invariably slight, or moderate in degree, when present, and that its presence should not affect unfavorably the prognosis.

*Abdominal distension.* It is noted whether the abdomen was, or was not distended in the histories of twenty-three cases. No distension was observed in nineteen of these twenty-three cases. In the remaining four cases, meteorism, or tympanites existed, but in each case it is noted to have been slight. Of the four cases in which meteorism was present, one only was a fatal case; and of the cases in which this symptom was absent, at least six were among those which proved fatal.

Thus it is evident that absence of abdominal distension is the rule in dysentery, with occasional exceptions in which there exists slight meteoric enlargement; and it is further evident that slight tympanites is no more an unfavorable, than its absence is a favorable indication.

*Condition of the tongue.* The histories of twenty-three cases contains information respecting the condition of the tongue. In three of these cases.

this organ presented no abnormal appearances. In the remaining twenty cases, coating existed in sixteen, and in four cases it was only formed or frosted, i. e. a change in color without an appreciable thickness of deposit on its superior surface. Dryness is noted in two cases, and it is stated that the tongue was moist in ten cases. It is noted in one case that the tongue was reddened, and in one case that the coating had a dark color.

Of the cases, the histories of which contain information respecting the tongue, four were fatal. Two of the three cases in which it is noted that the tongue presented a normal appearance, were among those which proved fatal. Of the remaining two fatal cases in one the tongue was coated, and in the other the coating had a dark color.

It is probable that in a large proportion of cases of dysentery the tongue presents morbid appearances. These appearances, however, have no special significance. They are common to this, and a host of affections. Practitioners who draw pathological inferences therefrom, must do so by a kind of divination, which is entitled to about as much confidence as belonged to the prediction of events, by the ancient augurs, from the flight of birds and an inspection of the entrails of animals. The same remark is applicable to the observation of the tongue in various, and indeed most other affections. A coating on the tongue is no indication of the severity of the dysenteric affection, since the organ not infrequently preserves a normal appearance, in cases ending fatally, and is observed probably as often, if indeed not oftener, in mild, than in grave cases.

*Thirst.* In the larger number of histories there is no reference to this symptom. It is noted to have been present in fourteen cases, but it is probable that it existed, to a greater or less extent, in many, if not most of the cases, the histories of which are deficient in information on this point. Of the fourteen cases in which it is noted to have been present, it was quite urgent, or extreme, in five; considerably so in two; moderate or slight in two, and in the five remaining cases, the degree of thirst is not stated. In two of the five cases in which the thirst was quite urgent, and one of the two cases in which it was considerably so, the disease proved fatal. The enumerations, however, are insufficient for any deductions respecting the bearing of this symptom on the prognosis.

*Appetite.* On this point, also, most of the histories are deficient in information. It is stated that the appetite was, or was not lost, in eleven cases only. Of these cases, in five anorexia existed, the appetite being impaired, but not lost in the remaining six cases. This would seem to show that anorexia existed in the smaller proportion of cases—a conclusion very probably incorrect. It would not be fair to judge the cases, the histories of which are silent on this point, by the results in the few cases in which the presence or absence of this symptom is noted. The presumption is, that the continuance of appetite would be more likely than its absence to arrest the attention, and thus be introduced into the histories, than as with respect to other symptoms which might be mentioned, their absence rather than presence would be deemed remarkable, and consequently be noted. All that is to be said under this head is, that in some cases of dysentery anorexia exists, and, in other cases, the appetite is retained, being more or less diminished. In one of the cases in which the appetite was not lost, the disease proved fatal, and as it happens, none of the five cases in which the appetite is noted as lost ended fatally. These facts suffice to show that anorexia may be present in mild cases, and that it is not uniformly present in those distinguished for gravity.

### SECTION FOURTH.

### *Symptoms referable to the circulation.*

Of the phenomena pertaining to the circulation, I have analyzed the cases with reference to the pulse only. In the great majority of the cases the pulse, during the progress of the disease, was increased in frequency. Of forty-two cases, the histories of which contain information on this point, in but four is it noted that there was no acceleration of the pulse. In the amount of acceleration the cases differed considerably. In some the frequency was not much greater than the average in health. In a small number, exclusive of the fatal cases, the increased frequency was great. In the case which presented the maximum of acceleration, (exclusive of the fatal cases,) the pulse was 140 per minute, for four successive days. At noon on the fourth day, it fell from 140 to 120, and during the next twenty-four hours it became reduced to 108. The patient was a female. Instances of even an approximation to so great frequency as this, were very few among the cases ending in recovery. In most of these cases the pulse was slightly, or only moderately accelerated, varying, in adults, from 80 to 100 per minute.

In the fatal cases, increased frequency of the pulse was uniformly noted, and the frequency was notably greater than in the cases ending in recovery. The two groups of cases, (fatal and not fatal,) are in a striking manner distinguished from each other by this circumstance. Selecting five fatal cases in which the enumerations of the pulse are recorded most fully, the mean frequency in the cases severally is as follows: Case No. 1. The enumerations commencing on the third day of the disease, 127. No. 2. Enumerations commencing on the second day, 119. No. 3. Enumerations commencing on the day of the attack, 123. No. 4. Enumerations commencing on the second day, 125. No. 6. Not stated on what day the enumerations were commenced, 112. These averages are considerably higher than in any five cases that might be seleeted from amongst those not fatal. Generally in the fatal cases the pulse became more and more frequent as the disease approached its termination, rising to 140, 150, 160, and becoming extinct for a greater or less period before death. One case furnished an exception to this rule. In this case on the day before that on which death took place, the pulse varied from 150 to 160; but on the day of death, and two hours prior to this event, it was appreciable. and had fallen to 132.

Judging from these observations the pulse is a good index of the gravity of this disease. If the pulse become notably accelerated, for example exceeding 120, in the adult, the patient is in considerable danger, and this danger increases, in a geometrical ratio, according as the pulse rises above the point just mentioned. A pulse of 140, unless it be of transient duration, although not absolutely a fatal symptom, points to an unfavorable issue. The probability of recovery, under such circumstances, is small.

The pulse, moreover, in some instances, indicates, by a rapidly increased frequency, an unfavorable change in the course of the disease occurring with equal suddenness. This fact was illustrated in several of the fatal cases in which, within a brief space, a striking difference in the frequency of the pulse was observed; sometimes this marked acceleration of the pulse succeeding another obvious event with which the change in the course of the disease was pathologically associated. As this is a point of practical importance, as well as interest, I will give the details in several of the cases, sufficiently to exemplify the fact just stated:

Case No. 1. In this case, up to the seventh day, the pulse was but slightly accelerated. On the eighth day it rose to 120, becoming, at the same time, small and irregular. Death occurred on the ninth day. The connection of the rapidly induced frequency of the pulse with any other event supposed to be concerned in the unfavorable change, is not noted in the history of this case.

Case No. 2. In the early part of the disease the pulse was but slightly accelerated. On the sixth day it rose to 140. On the seventh it fell to 120, and on the eighth to 116. On the evening it rose again to 140, and afterward to 160. The association of these variations with other pathological events by which they may be accounted for, is not noted.

Case No. 3. In this case, a week after the date of the attack, the pulse was 120. In a few hours, a copious sero-sanguinolent evacuation having in the mean time occurred, it rose to 140. Death on the following day.

Case No. 4. When the patient came under observation, on the second day of the disease, the pulse was 125. It fell, on the following day, to 112. On the next day, copious sanguinolent dejections having in the mean time occurred, it rose to 150, and became nearly extinct on the next day. Death occurred on the day subsequent to the latter.

Case No. 5. On the first and second days after the attack, the pulse was 100. On the third day, 120 at A. M. and 100 at P. M. On the fourth day, at A. M., 116, at evening, 124; later, 140; later still, 150. On the fifth day, 160. On the sixth, day of death, extinct. In this case, sero-sanguinolent dejections occurred more or less frequently from the commencement. The rapid increase in the frequency of the pulse, on the fourth day, however, was not associated with a remarkable increase in the copiousness of this discharge, or any other obvious event accounting for the rapidly developed change for the worse.

Case No. 6. For the first two days the pulse was not accelerated. On the third day, for the first time, the evacuations were sero-sanguinolent. The pulse on that day became 120, and at evening, 125. On the fifth day, at morning, the pulse was 120; at noon, 116; at 9½, P. M., 120; at 11½, P. M., a copious sero-sanguinolent dejection having taken place, 135. Death occurred four days afterward.

A rapidly accelerated pulse occurring in the connection of sequence with abundant sero-sanguinolent discharges, is a fact worthy of notice, but this connection is not in all instances to be observed. A notable frequency of the pulse taking place suddenly, however, should always be regarded as significant of some very unfavorable pathological change, although the character of the change may not be understood. In some cases the pulse is the chief index of such a change. It, therefore, becomes of great importance sometimes in giving the first warning of the existence of danger, and of its extent. No other source of symptoms, perhaps, in this disease, has a more important bearing on prognosis.

It is to be borne in mind that, although a marked acceleration of the pulse attends this disease, probably, in the majority of cases, when it assumes great severity, yet, in some instances in which the disease proceeds to a fatal issue, the increase of the pulse is at no time great. This important fact is illustrated by a few of the fatal cases in this collection.

In the case No. 1, before referred to, the pulse did not rise above 120. In another case the pulse was only moderately accelerated, the enumerations not being given. In another case it did not exceed 112.

Thus, we are by no means warranted in considering a patient free from danger, although the pulse may not be accelerated more than obtains in many cases which end in recovery.

Of the characters pertaining to the pulse, aside from its frequency, it will suffice to speak, in general terms, without giving details. In a small number of cases, in the early part of the disease, the pulse being moderately accelerated, it had considerable development and force. This kind of pulse was associated with heat of surface; in other words marked febrile reaction, or symptomatic fever was present. Generally, however, when the pulse was accelerated, its quality denoted irritability, rather than increased power. If considerably accelerated, it was either feeble and small, or it was jerking, vibratory, and compressible; and these evidences of diminished power in the forces carrying on the circulation were in proportion to the increase in frequency. In the fatal cases in which there was not great acceleration of the pulse it was small and feeble. As already stated, in most of the fatal cases the pulse became extinct for some time before death. Usually death occurred in a few hours after the pulse became inappreciable, but in one instance life was prolonged over twenty-four hours after the pulse had ceased to be felt at the wrist.

### SECTION FIFTH.

### *Symptoms referable to the nervous system.*

The only symptoms referable to the nervous system which enter into the histories, are pain, and aberrations of the mind. As respects the former, the pain was in every instance confined to the abdomen, and the facts concerning this symptom have been sufficiently noticed already, under the head of tormina, in section third. It remains only, therefore, in the present section, to present the facts which pertain to the mental condition.

In not one of the cases ending in recovery is the presence of delirium noted. Among the cases proving fatal, a striking contrast was exhibited as regards this symptom. In some the intellect was retained with remarkable clearness to the close of life; in other cases delirium existed in a marked degree. Of nine cases, the histories of which are complete in this particular, in four the patients preserved clearness of mind up to the last moments. In the remaining five cases more or less delirium existed. In two of these five cases the mental aberration consisted only in slight wandering, indicated by incoherency, coming on toward the close of life. In the other three cases the delirium was active and constituted a prominent symptom. The patients in these cases were boisterous, attempted to get out of bed, and required restraint. In one of the cases so violent were the efforts, at a time when the other symptoms denoted impending dissolution, accompanied by loud talking and singing in a manner painfully incongruous, that it was deemed advisable to resort to chloroform, and the patient was kept under its influence in a degree sufficient to enforce quietude for eight or nine hours preceding the fatal termination.

The striking contrast thus exhibited among the fatal cases is an interesting pathological fact. It would appear that sometimes the dysenteric affection is directed toward the cerebral organs, while at other times, this portion of the organism is singularly exempt from any participation in the morbid processes involved in the disease. We cannot offer any explanation of this discordancy without the aid of speculative reasoning, and this would be out of place in this report in which the writer aims to keep strictly within the limits of historical facts, and the pathological conclusions to be legitimately deduced therefrom.

So far as the present collection of cases furnishes data on this point, it would follow that delirium is, to say the least, an event extremely unfavorable as respects prognosis, in dysentery, since it was alone observed among the cases which proved fatal. It may be, however, that were the number of cases considerably larger, a different conclusion might be deducible. Past observations by different persons, lead to the supposition that the occurrence of delirium, as a prominent feature of this disease, characterizes its prevalence, as an epidemic, at certain times and places. Systematic writers have instituted a variety of the affection denominated typhous dysentery, of which a point of delirium analogous to that of the typhus and typhoid fevers is a distinctive trait. This title implies an hypothesis which it would not be appropriate to discuss in this connection. The relations which the symptom under consideration, (delirium,) bears, not alone to the prognosis, but to the pathological character of the disease, is one of the subjects, with reference to which more extended analytical investigations are desirable. Although active delirium was not present in any of the cases ending in recovery, among those now analyzed, it has fallen under my observation when the issue was favorable. In a case, of which no notes were made, this symptom was developed early, and continued, in a notable degree, during the whole career of the disease. The dysenteric discharges were severe, copious sero-sanguinolent and mucous evacuations taking place.

The gravity of the disease was greater than in any other case, ending in recovery, that has ever fallen under my observation. In conclusion, we are, perhaps, justified in saying that the occurrence of delirium as a prominent symptom in dysentery, exclusive of epidemics in which it is present as a characteristic, must be regarded as an extremely unfavorable event, and should lead us to anticipate a fatal result.

### SECTION SIXTH.

### *Symptoms referable to the skin.*

*Skin.* In a portion only of the histories (20) is the condition of the skin noted. An examination of the facts observed in these cases, shows sweating to be an event occasionally occurring in the course of this disease. It occurred in several of the fatal cases toward the close of life; in other instances the skin remaining dry. Aside from its occurrence under these circumstances, it was noticed in but a small proportion of cases. It is not specially significant of danger, unless, at the same time, other symptoms denote a change for the worse, since the not fatal cases in which it was observed were not particularly severe, and some were mild. Moisture of the surface, that is, a degree of perspiration falling short of sweating, is noted in a few cases. It is very probable that this occurred in more or less of the cases in the histories of which no reference is made to it. It would be more likely to happen that moisture would not be noted, than that sweating would escape attention in recording the histories. In a certain proportion, however, of cases of dysentery, perhaps in the larger number of those ending in recovery, the surface remains free from sweating or moisture.

As regards temperature, in most of the cases the skin was cool, or retained the normal degree of heat. In a few instances an increase of temperature was observed. This was in the early part of the disease forming an element of the febrile reaction which, as already stated, was observed in a small number of cases. In the fatal cases, coldness of the surface uniformly preceded, for a greater or less period, the termination of the disease; the coldness sometimes accompanied by clammy moisture or sweating, and in other instances the skin remaining dry, the body, for some time prior to death, feeling like a cadaver.

None of the eruptions described by authors as incident to dysentery, were observed in the cases under analysis.

### SECTION SEVENTH.

#### *Symptoms referable to the respiratory system.*

I have analyzed the histories with reference to morbid phenomena pertaining to the respiratory system, but, with a very few exceptions, they contain nothing of importance falling under this head. It is noted in the history of one of the fatal cases, that the respirations were abnormally infrequent, becoming more and more slow as death approached. In but one instance, only, is it recorded that cough and expectoration were present. In this case the patient stated that she had taken cold just before the dysenteric attack. The symptoms just mentioned were, in this instance, slight. One patient complained of a painful sense of the want of breath for several hours before death. These are all the facts contained in the histories belonging to this division of the analytical investigation.

It is evident that the pulmonary apparatus does not furnish morbid elements entering into the natural history of dysentery. Or, to vary the language, the intimate pathological condition which constitutes this disease, so far as a judgment may be based on its symptomatology, has no special manifestations in that portion of the organism.

### SECTION EIGHTH.

#### *Duration of disease to death or convalescence. Mode of dying. Relapses. Fatality.*

I will examine the facts with respect to the duration of the disease, etc., *first*, in the cases which ended in recovery; and, *second*, in those terminating fatally.

*Duration, etc., in the not fatal cases.* The duration is dated from the commencement of the illness, not from the time when the dysenteric discharges were first observed. It has been seen, (section second,) that the characteristic evacuations were usually preceded for one, two, three days, or more, by ordinary diarrhœa. It is, perhaps, proper to consider the disease as having commenced when the latter was present, and the duration is determinable in a larger number of cases, by pursuing this course. The ending of this disease is fixed at the time when the dysenteric discharges ceased entirely, or there was such a marked improvement in all the symptoms that the patient could be pronounced convalescent. In determining the latter boundary, some latitude of judgment is of course allowable. The duration cannot be ascertained with rigorous exactitude, but sufficiently so for all practical purposes. The data for determining the precise duration, or a close approximation thereto, are contained in thirty of the thirty-eight cases that ended in recovery. The periods varied considerably in the different cases. The shortest duration was a single day, but this was true of but one case only. The next shortest period is four days. In one instance only was the career of the disease so short as this. In four cases the disease lasted but five days. In two cases it lasted six days; in three cases seven days; in four cases eight days; in one case nine days; in four cases ten days; in one case eleven days; in two cases, twelve days; in two cases, thirteen days; in two cases, fourteen days; in no instance fifteen days; in two cases, sixteen days; in one case twenty-one days, and in no instance for a longer period.

The mean duration in this group of cases, is nine 5-6 days.

A striking disparity is developed by a comparison of the duration in the cases occurring in private practice, with those treated in hospital. Of the thirty cases, thirteen were in hospital, and seventeen in private practice. The mean duration in the thirteen hospital cases, is just thirteen days. Of the seventeen cases in private practice, the mean duration is only a fraction over seven days! The explanation of this difference which suggests itself is, that the hospital patients were generally admitted after the disease had existed for some time, frequently without having received any treatment, and sometimes, perhaps, having been injudiciously treated. That the condition of the hospital patients after admission was even better than that of the patients in private practice, is rendered probable by the fact that the ratio of fatality among the latter was considerably greater. Of twenty-seven patients in private practice, eight died; while of twenty-two hospital cases, in only three was the disease fatal. It is a curious fact that the duration of the disease should be nearly twice as great in the hospital cases, and at the same time the mortality less than one-half in these cases, contrasted with those occurring in private practice! The fact proves that, whatever were the causes which occasioned the longer duration in the patients treated in hospital, they were not of a character to increase the danger of a fatal result; and it is a gular conclusion, which at first view appears paradoxical, that there may exist causes tending to prolong this disease, while, if the tendency to a fatal result be at all influenced thereby, it is lessened rather than increased by them! It serves to reconcile this apparent incongruity of the duration and fatality, to suppose that the situation of the patients in hospital was more favorable for the successful treatment of the disease than that of the patients in private practice.

The advent of convalescence was denoted by cessation, or marked diminution of the dysenteric evacuations, and a return of their normal appearanec, an amelioration of the symptoms generally taking place at the same time. The rapidity with which the natural feces reappeared, varied considerably in different cases. In some the mucus and bloody dejections ceased almost at once, but in other instances they disappeared more gradually, and in a few cases these morbid appearances were occasionally observed, for several days after the patients were decidedly convalescent. In a very small number of cases only did the affection continue and assume a chronic character. This was true only of two of the cases analyzed, and in these cases recovery took place after a few weeks. Thus, in not a single instance among the cases in this collection, was that obstinate form of chronic dysentery now and then met with in medical practice produced as a sequel of the acute disease. This fact, coupled with the infrequency of cases of chronic dysentery so far as my experience goes, leads me to infer that the intestinal affection, except in the instances in which the severity of the disease leads to a fatal result within a short period, tends to complete recovery in the great majority of cases.

Nor were relapses observed. In not a single instance did a repetition of the dysenteric affection occur after convalescence was pronounced. This is an interesting fact, taken in connection with some other facts, in its pathological bearing; going to show that the disease belongs in the category with those which involve certain processes (zymotic) within the organism, reproduced with difficulty after they are completed.

Finally, the recovery from the disease, in the great majority of cases, was rapid, as well as complete.

*Duration, etc., in the fatal cases.* The duration is determinable in ten of the eleven cases forming this group. The number of days from the date of the attack to the time of death, in the cases respectively, are as follows: six days, two cases; seven days, two cases; nine days, two cases; ten days, two cases; eleven days, one case, and nineteen days one case.

The mean period is a fraction over nine days.

The brief duration accords with what was stated with respect to the tendency of the disease to become chronic. So far as a judgment may be based on the data contained in this collection of cases, the affection is one which usually runs on, rather rapidly, either to a fatal termination, or to recovery. In this respect it resembles the essential fevers. The pathological significance of this fact is obvious.

The mode of dying was in every instance by asthenia. The progress toward a fatal termination was marked by progressive diminution of the forces carrying on the circulation, shown by the pulse becoming more and more feeble, generally proportionably frequent, and finally becoming extinct, the patient falling into a state of collapse. The rapidity with which the state of collapse was induced, and the period which afterward elapsed before death, varied considerably. The connection which was observed between the induction of this state, or of a notable change in the circulation eventuating therein, and the occurrence of copious sero-sanguinolent discharges has been already referred to in section third. Also the striking difference as regards the condition of the intellect in the latter part of the disease in the fatal cases has been noticed in section fifth.

*Fatality.* Of the forty-nine cases analyzed, eleven proved fatal. Adding to the forty-nine cases the eleven which were rejected in consequence of too great deficiencies in the histories, of the whole sixty cases, thirteen proved fatal. This does not show the ratio of fatality to the whole number of cases treated by me during the period that these histories were collected, for, during this period quite a number of cases were observed of which no notes were taken, while, my belief is that the thirteen fatal cases just mentioned, include all that have fallen under my observation in which recovery did not take place. But it would be of little use to determine with exactness the total number of deaths to the whole number of cases, for an examination of the relative number of deaths in different periods of the whole time over which the dates of the cases in this collection extend, will show that the tendency of this disease to a fatal issue is by no means uniform. It is with reference to the latter point, chiefly, that the facts contained in the present collection of cases possess any interest. To this point, therefore, the interrogation of the histories will be restricted.

The first of the fatal cases among those analyzed, occurred in July, 1848. In this case I was not the attending physician, but visited in consultation. The first fatal case which ever came under my observation, in which the responsibility of the management devolved solely upon me, was in July, 1849.

This is the fifteenth case in the present collection, numbered in chronological order. In the year 1849, dysentery prevailed in this city, as an epidemic, in connection with epidemic cholera. All the cases that I had observed prior to this time, were sporadic cases, and all recovered. I had never witnessed a fatal issue of the disease. During the summer and autumn of 1849, I recorded the histories of twenty-six cases. Of these twenty-six cases four were fatal. The eleven remaining cases occurred during the years 1850, 1851, and 1852. Of these eleven cases six were fatal. Dividing the whole time during which the histories were collected, into the three periods just referred to, we have 1st. The period of eight years when dysentery occurred only as a sporadic disease, and cases were very infrequent; 2d. The year of 1849, in which it prevailed considerably as an epidemic following cholera; and 3d. The three subsequent years, when it prevailed as an epidemic, but to a much less extent than in 1849. Now I cannot present the exact ratio of fatal cases to the whole number observed during these periods respectively, because in each period a number of cases ending in recovery fell under observation which were not recorded. But, assuming that the number of recorded cases in the three periods, represents the relative prevalence of the disease in the periods respectively, an assumption which I am satisfied is not far from the truth, and the facts pertaining to the fatality show, *first*, the absence of a tendency in the disease in this situation to a fatal issue when it occurs as a sporadic affection; and, *second*, that as an epidemic, the inherent tendency, in the same situation, is greater at some seasons than at others. These conclusions are by no means novel. Moreover, I am free to admit that they are not rigorously proved. An important postulate in the argument rests only on opinion. It is necessary to assume, also, that the management, in the three periods, was the same, or equally judicious; and the number of fatal cases is too small to be independent of coincidences. With these qualifications the deductions would not be entitled to much consideration were they opposed to views generally entertained; but the fact that they go to sustain these views is presumptive evidence of their correctness.

### SECTION NINTH.

### *Supposed causative agencies.*

By the above caption I do not mean to refer to the occult, special cause, or causes which it is probable are necessarily involved in the production of dysentery, but to any obvious circumstances, which, occurring at, or near the time of the attack, might be suspected to have exerted some agency in the development of the disease. In other words, it is chiefly with reference to exciting, not essential causes, that the histories may be expected to contain historical information. The analysis reveals scarcely any thing of importance falling under this head. Among the whole forty-nine cases, in but two instances are any circumstances stated to which the disease appeared, in any measure, to be attributable. In one of these cases the patient had eaten freely of green fruit the day before the attack, and it is noted, also, that at the time of the attack, a considerable change in the weather had taken place, from hot to cold. In the other case, the patient had eaten freely of ice cream, and drank champagne wine the evening prior to the day of the attack.

In a few of the histories it is noted that there were no circumstances which might be suspected to stand to the disease in the relation of exciting causes, but generally nothing is recorded on the subject. It may be thought that, in the cases in which the records are silent, attention was not directed to this point, and that perhaps particular inquiries would frequently have elicited information bearing on the causation. I admit it would have been more satisfactory had the histories declared that such inquiries had been instituted with negative results; but it may be remarked that patients are usually very ready to assign some circumstance, or circumstances as the causes of their illness, and that circumstances are more apt to be voluntarily communicated, (with certain obvious exceptions,) relating to this, than to any other point. It is, therefore, probable, that in most, if not all the cases in which nothing is noted concerning the causation, nothing had occurred which, in the patients' estimation, occasioned the disease. Reasoning in this way, the inference to be drawn from the negative facts in this collection of cases is, that dysentery, in the great majority of cases, does not involve any obvious exciting causes. This conclusion has more significance than, on a superficial view, may appear. In proportion as any affection is shown to be independent of apparent external circumstances is it evident that its causation involves inappreciable and special morbid agencies. This may be stated as a law of etiological investigation. And, in accordance with this law, the absence of exciting causes, as the rule, is evidence that dysentery proceeds from the operation of influences to be included in the class of those which have, as yet, eluded scientific research.

In one of the cases the disease occurred shortly after recovery from epidemic cholera. The latter affection may have predisposed to the former.

In another case the patient was attacked with a mild form of the disease, which, on the third day, was so far relieved that it was anticipated he would be able to go out on the following day, when an officious neighbor persuaded him to be stripped and undergo a process of ablution in a tub of hot water containing mustard. The next morning the attack was renewed with great severity, and the disease proved fatal.

As already stated, our present range of inquiry does not embrace the subject of the special or essential agencies involved in the causation of dysentery. Some facts, however, have already been submitted, (section first,) which have an important bearing on the question whether the disease does involve such agencies, or not. Reference is had to the influences incident to season and to different years. The consideration of these, and other circumstances bearing on this subject, will come up in a more appropriate connection after the completion of the clinical report.

In conclusion, my observations fail in supplying any facts going to show the contagiousness of dysentery, a doctrine which, as is well known, has heretofore had its supporters, and which circumstances have sometimes seemed to sustain. Whether the disease in any of its forms or associations may not be communicable, cannot, of course, be settled by the data which I have collected; but, if ever contagious, it has certainly not given any evidence of this property in the cases, sporadic or epidemic, that have fallen under my observation.

### SECTION TENTH.

### *State of health subsequent to recovery. Recurrence of the disease.*

The subjects of this section are interesting, and so far as I know, have not been investigated by means of recorded facts. What remote effects, if any, in the organism, follow an attack of dysentery? Does it beget a predisposition to any other disease? Does it impair the constitutional vigor? Is the patient afterward liable to renewed attacks of the disease, or is the susceptibility to the action of the cause, or causes, lessened, or destroyed, so that the morbid processes having once taken place, there is greater security, if not entire exemption thereafter? These are questions interesting in themselves, and important in their pathological bearings. The facts in this collection of cases pertaining to these subjects are not numerous, but few as they are, they lead to the belief that they involve something more than coincidences: that they point to laws of the disease which do not appear, as yet, to have received attention. I will proceed to present the results developed by the analysis.

Of the thirty-eight cases ending in recovery, in sixteen the patients either continued under observation, or definite information was obtained respecting their subsequent health for periods varying from one year to thirteen years. To be more precise, with regard to these periods, the duration of the subsequent history in the cases respectively are as follows: thirteen years, one case; seven years, two cases; five years, two cases; four years, nine cases; two years, one case, and one year, one case.* In all, sixteen cases. Of these sixteen patients one only has died. This patient died with tuberculosis of the lungs, four years after recovering from dysentery. Of the remaining fifteen cases, in all the health has been good during the periods, respectively, during which the patients remained under observation, or within knowledge. I mean by this, that not one has suffered from any other important disease, and in nearly every instance there has been excellent health.

*Not one of these fifteen patients has experienced a second attack of dysentery.* This result is rendered somewhat more striking by the fact that, exclusive of the case in which the date of the disease was but a year since, all the patients have been for several years past within the sphere of an epidemic influence, the disease, as already stated, having prevailed more or less, as an epidemic, in this city, in the autumnal months for the last four years. I can also call to mind several cases of dysentery occurring at least four years since, the histories of which were not taken, the patients, in the mean time, being under observation. In none of these instances has the disease been twice experienced. *I have not yet met with a second attack of dysentery.* It is worthy of notice that three of the cases in this collection occurred in one family at different dates, viz., one case, thirteen years ago; one four years, and one two years. In these instances new subjects were attacked, they escaping who had previously had the disease.

It would seem, from these facts, that an attack of dysentery does not exert an unfavorable influence on the organism; that patients are not rendered thereby prone to any particular disease, but, on the other hand, enjoy good health. It would also seem that patients are not liable to a repetition of the disease.

I would not be understood as presenting these inferences in the light of established truths. The number of observations is too small to warrant deductions which shall represent fixed laws of the disease. But, as already remarked, it is difficult to account for the facts on the hypothesis of their being due to mere coincidence; and I repeat that the facts point to the

* I do not give the periods with more exactness than the number of years. In no case does the period fall short of the year specified; in several cases it extends some months longer.

existence of laws which remain to be settled by farther investigation. We are at least justified in concluding, from the data before us, that deterioration of the health subsequent to dysentery is not the general rule, and that an attack does not lead to any increased susceptibility to the cause, or causes of the disease.

NOTE. — The conclusion drawn from the facts developed by this analysis with respect to the recurrence of the disease, I am aware is at variance with authority on this point. Valleix, for example, says, "On a également noté assez fréquemment de véritables *récidives;* car la dyssenterie n'est pas une de ces affections qu' on n' éprouve qu' une seule fois dans la vie. Certains sujets y sont particulièrement exposés, et en sont fréquemment atteints." * He does not, however, adduce facts in proof of this statement: but I am not prepared to declare that the statement has not been proved by reported facts.

(To be continued.)

* Guide du Médicin Praticien. Tom. Troisième.

